# Widefield *en face* optical coherence tomography monitoring of the peri-venular fern-like pattern of paracentral acute middle maculopathy

**DOI:** 10.1016/j.ajoc.2021.101047

**Published:** 2021-03-10

**Authors:** Prashanth G. Iyer, Swarup S. Swaminathan, Omer Trivizki, Yingying Shi, Mengxi Shen, Mary Kansora, Giovanni Gregori, Philip J. Rosenfeld

**Affiliations:** a Department of Ophthalmology, Bascom Palmer Eye Institute, University of Miami Miller School of Medicine, Miami, FL, USA; b Veterans Affairs North Texas Health Care System – Dallas, UT Southwestern Medical Center, Dallas, TX, USA

**Keywords:** Paracentral acute middle maculopathy (PAMM), Peri-venular fern-like PAMM, en face structure OCT, Ischemic cascade, Retinal artery occlusion

## Abstract

**Purpose:**

To demonstrate the ability of widefield *en face* swept source optical coherence tomography (OCT) imaging to monitor peri-venular fern-like patterns of paracentral acute middle maculopathy (PAMM) associated with retinal arterial occlusions.

**Observations:**

The peri-venular fern-like pattern of PAMM was diagnosed on the 12 × 12 mm *en face* structural OCT images from three patients. Fluorescein angiography images were unremarkable. Over time, all three patients demonstrated significant improvement in visual acuity with resolution of their peri-venular PAMM.

**Conclusions:**

The peri-venular fern-like pattern of PAMM is usually associated with retinal vein occlusions, but we identified three cases with this pattern in eyes with presumed incomplete retinal arterial occlusions. Our cases support the ischemic cascade theory that begins within the deep capillary plexus and ascends in the retina depending on the severity of the ischemic event. Using the 12 × 12 mm *en face* structural OCT images, we are able to demonstrate a wider area of ischemia in PAMM compared with the traditional 6 × 6 mm scans.

## 1 Introduction

Paracentral acute middle maculopathy (PAMM) is a finding seen on optical coherence tomography (OCT) imaging that corresponds to ischemia in the deep and intermediate retinal capillary plexi.^1^, ^2^, ^3^, ^4^ A hyperreflective band can be seen in the inner nuclear layer on OCT.^4^ PAMM can be associated with conditions such as diabetic retinopathy, hypertensive retinopathy, retinal arterial and vein occlusions, sickle cell retinopathy and other ischemic conditions.^1^, ^2^, ^3^ Fundus examination and fluorescein angiography (FA) findings may be unremarkable, but OCT imaging can reveal PAMM that suggests a serious underlying pathology and may warrant further investigation and systemic workup. While vision may not be significantly affected, patients may complain of a paracentral scotoma and visual distortions.^4^

PAMM can be appreciated on *en face* OCT imaging by looking at a slab in the middle of the retina that includes the inner nuclear layer and the deep retinal capillaries. Three patterns of PAMM have been identified which include the peri-venular fern-like, arteriolar, and globular patterns.^3^ These patterns have been thought to reflect the spectrum of this disease, with the peri-venular fern-like pattern indicating milder disease and the globular pattern indicating more severe ischemia. The peri-venular fern-like pattern, which is characterized by lesions surrounding veins with peri-arterial sparing, has generally been associated with retinal vein occlusions.^5^^,^^6^ Swept source OCT (SS OCT; PLEX Elite 9000, Carl Zeiss Meditec, Dublin, CA) can offer an advantage over traditional OCT imaging because of its wider field of view (FOV). We present three unique cases of peri-venular fern-like patterns of PAMM imaged using the 12 × 12 mm scan pattern obtained using SS-OCT.

## 2 Cases

### 2.1 Case 1

**Fig. 1:**
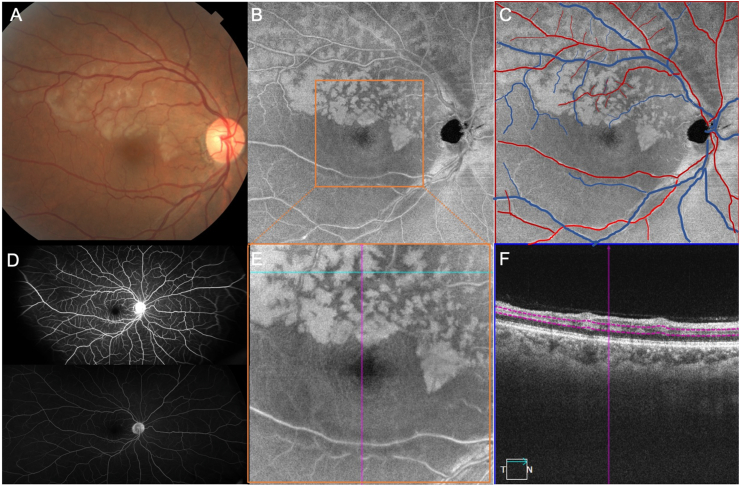
Case 1: Color fundus imaging, widefield *en face* imaging of the middle retina, and fluorescein angiography of a branch retinal artery occlusion. (A) Color fundus image of the right eye shows retinal whitening along the superior arcade extending to the fovea, representing a right branch retinal artery occlusion. (B) *En face* structural optical coherence tomography (OCT) image of the middle retina from a 12 × 12 mm scan demonstrates a peri-venular hyperreflectivity in a fern-like pattern. (C) Retinal vascular map corresponds to the peri-venular hyperreflectivity, with peri-arterial sparing. (D) Fluorescein angiography (FA) of the right eye at early and late stages does not reveal any areas of ischemia or transit delays. (E) 6 × 6 mm *en face* structural OCT image with vertical and horizontal navigation lines (purple and blue) showing the area between non-ischemic and ischemic retina, with similar findings on the corresponding B-scan around the inner nuclear layer in a skip pattern (F). (For interpretation of the references to colour in this figure legend, the reader is referred to the Web version of this article.)

A 48-year-old Asian woman with a past medical history of hypertension presented with one day history of persistent blurry vision in the right eye. She had two previous episodes of transiently blurred vision in the right eye that resolved spontaneously. Visual acuity (VA) of the right eye was 20/70 + 1. Examination revealed retinal whitening along the superior arcade extending to the fovea (Fig. 1A), consistent with a superior branch retinal arterial occlusion (BRAO). *En face* structural OCT images of the middle retina from the 12 × 12 mm scan showed a peri-venular hyperreflectivity in a fern-like pattern (Fig. 1B) that corresponded to the retinal vascular map (Fig. 1C). Early and late FA images of the right eye did not reveal any areas of retinal nonperfusion (Fig. 1D). On closer examination of the macula using 6 × 6 mm *en face* structural images, the junctional zones between the presumed regions of perfused and ischemic retina could be easily identified (Fig. 1E) with the ischemic regions corresponding to areas of hyperreflectivity seen on the corresponding B-scan (Fig. 1F). The patient was admitted for a full cardiac workup. At one-month follow-up, there was improvement of the PAMM on *en face* structural OCT images and corresponding B-scans (Fig. 4A–C). Five months later, the BRAO had resolved with residual inner nuclear layer thinning and visual acuity in the right eye was 20/20.

### 2.2 Case 2

**Fig. 2:**
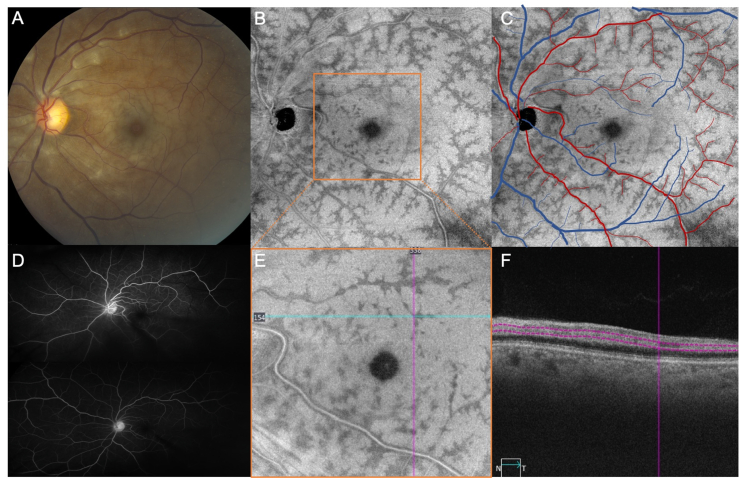
Case 2: Color fundus imaging, widefield *en face* imaging of the middle retina, and fluorescein angiography of a central retinal artery occlusion. (A) Color fundus image of the left eye shows diffuse retinal whitening, consistent with a left central retinal artery occlusion. (B–C) *En face* structural OCT image from the middle retina of the 12 × 12 mm scan shows a peri-venular pattern of PAMM around the retinal veins, sparing the retinal arteries. While fluorescein angiography does not show any transit delays or nonperfusion (D), the junction between the ischemic and non-ischemic retinal tissue is demonstrated on 6 × 6 mm *en face* structural OCT (E) and B-scan using the slab between the inner nuclear layer in a skip pattern (F). (For interpretation of the references to colour in this figure legend, the reader is referred to the Web version of this article.)

A 59-year old African-American man with a past medical history of hypertension, hyperlipidemia, and cerebrovascular accident presented with sudden onset blurry vision of the left eye. VA of the left eye was 20/60. Fundus examination revealed diffuse retinal whitening in the parafoveal region and along the vascular arcades consistent with a central retinal artery occlusion (CRAO (Fig. 2A)). *En face* structural OCT images of the middle retina from the 12 × 12 mm scan revealed a peri-venular pattern of PAMM around the retinal veins and sparing the retinal arteries (Fig. 2B–C). While FA did not show any transit delays or nonperfusion (Fig. 2D), the areas of ischemic and non-ischemic retina seen on the *en face* slab of the inner nuclear layer and the B-scan images were consistent with PAMM (Fig. 2E–F). At that visit, the patient was admitted for a stroke and cardiac workup. Over a two-month period, there was improvement in the peri-venular PAMM outside of the central macula (Fig. 4D–F). B-scans revealed thinning of the temporal foveal contour and retina (Fig. 4F). With time, retinal whitening on examination resolved with residual retinal thinning and the patient's acuity stabilized at 20/40.

### 2.3 Case 3

**Fig. 3:**
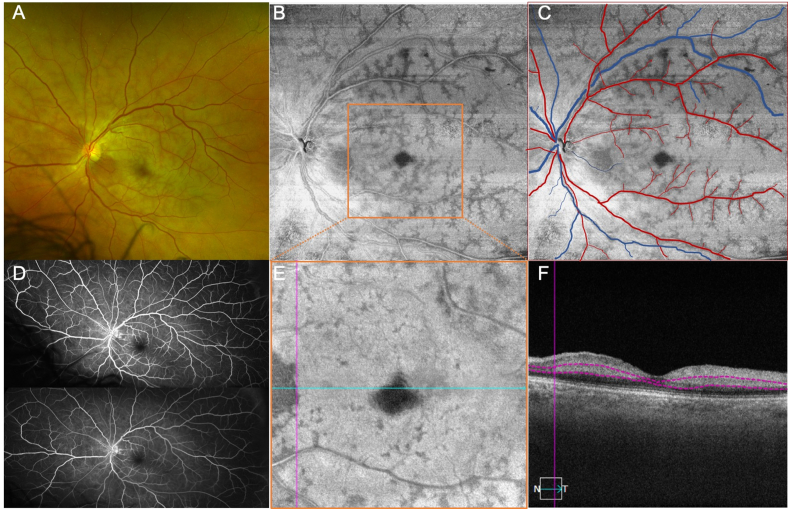
Case 3: Color fundus imaging, widefield *en face* imaging of the middle retina, and fluorescein angiography of a central retinal artery occlusion (A) Color fundus image of the left eye shows diffuse retinal whitening around the posterior pole, consistent with a central retinal artery occlusion. (B) *En face* structural OCT images of the middle retina from the 12 × 12 mm scan shows the peri-venular fern-like pattern of PAMM, confirmed by the retinal vascular map (C). (D) Fluorescein angiography does not show any transit delays or nonperfusion. (E–F) On closer examination using 6 × 6 scan of the *en face* structural OCT, the junction between ischemia and non-ischemia is seen and corresponds to the B-scan. (For interpretation of the references to colour in this figure legend, the reader is referred to the Web version of this article.)

**Fig. 4:**
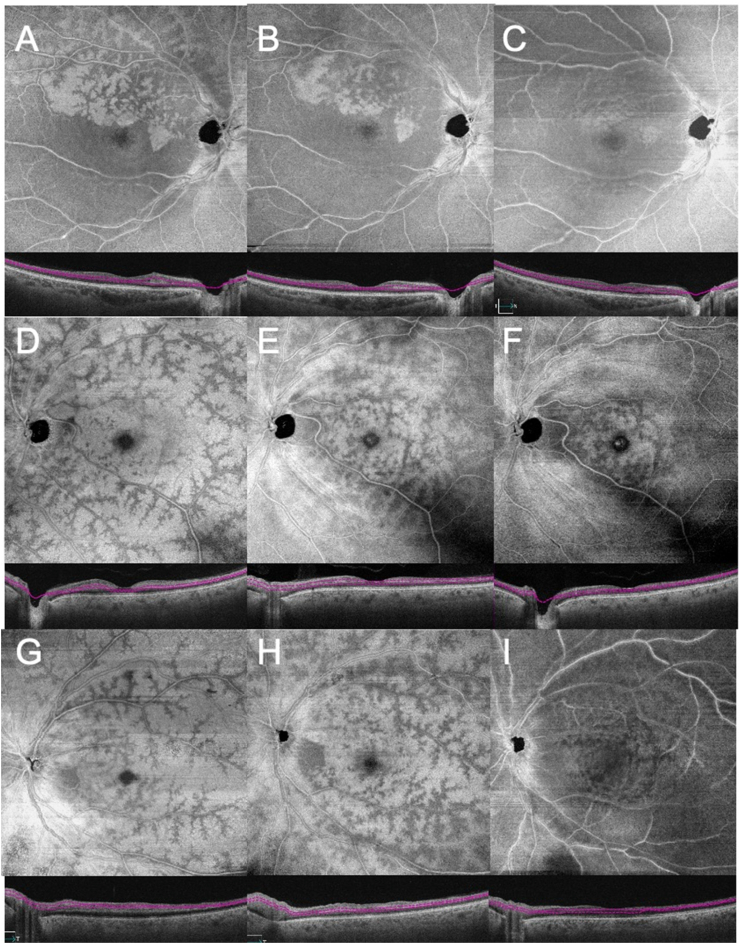
Follow-up images of Cases 1–3 using *en face* 12 × 12 mm scan OCT images of the middle retina demonstrating regression of the peri-venular fern-like pattern over time. (A–C) Patient with right branch retinal artery occlusion at presentation (A), shows improvement of PAMM at one week (B) and over one month (C). (D–F) Patient with left central retinal artery occlusion reveals reduction of ischemia at two weeks (E) and over two months(F). (G–I) Patient with left central retinal artery occlusion demonstrates reduction of ischemia at one week (H) and one month (I).

A 67-year-old African-American man with a past medical history of diabetes mellitus, hypertension, hyperlipidemia, and obstructive sleep apnea, presented with sudden blurry vision in his left eye. VA of the left eye was 6/200, complicated by a chronic dense nuclear sclerosis. Fundus examination revealed diffuse retinal whitening around the posterior pole (Fig. 3A) consistent with a CRAO. *En face* structural OCT images of the middle retina from the 12 × 12 mm scan demonstrated the peri-venular fern-like pattern of PAMM (Fig. 3B) confirmed by the retinal vascular map (Fig. 3C). FA did not show any transit delays or nonperfusion (Fig. 3D). On closer examination using the *en face* structural OCT image and B-scan from the 6 × 6 scan, the junction between ischemic and non-ischemic retina could be appreciated (Fig. 3E–F). At the one-month follow-up visit, the VA was 20/70 with a potential acuity meter measurement of 20/40. Examination showed resolution of the retinal whitening and *en face* OCT images showed significant improvement of the peri-venular fern-like PAMM with residual inner nuclear layer thinning (Fig. 4G–I).

## 3 Discussion

PAMM is an OCT finding that can be used as a tool to detect retinal vascular events even when clinical examination or FA are unremarkable. PAMM affects the inner nuclear layer, which is flanked by the intermediate capillary plexus (ICP) and deep capillary plexus (DCP).^1^, ^2^, ^3^, ^4^ Depending on the severity and the type of the underlying retinal condition, distinct patterns of PAMM can present on structural OCT imaging, and they include the globular, arteriolar and fern-like peri-venular subtypes.^3^ In this current case series, we present three cases of peri-venular fern-like pattern of PAMM in the setting of a presumed retinal artery occlusion, which demonstrates that peri-venular PAMM can occur in a range of ischemic events, not just retinal vein occlusions. The 12 × 12mm scan pattern corresponds to a 40-degree field of view (FOV) that doubles the 20-degree FOV offered by the more typical 6 × 6mm scan pattern and is acquired in nearly the same amount of time. Moreover, the 12 × 12mm scan shows that the manifestations of PAMM can extend outside the paracentral location that was initially described. If 12 × 12mm scans had been available when first described, then PAMM may have been defined as macular acute middle maculopathy or MAMM. Peri-venular PAMM could be seen on widefield *en face* structural OCT images in all of our cases despite no fluorescein angiographic correlations, suggesting that *en face* structural OCT imaging may be a superior imaging modality in PAMM compared with FA.

All three cases demonstrated improvement of the fern-like pattern over time. By using the *en face* images from the 12 × 12 mm scans, we were able to show that PAMM extended over a larger area than the usual FOV provided by a 6 × 6 mm scan and the diagnosis of PAMM was immediately apparent from looking at the extent of the fern-like pattern on the 12 × 12mm scan. With one non-invasive imaging modality, the *en face* structural OCT image using the 12 × 12 mm scan not only detected the peri-venular fern-like pattern of PAMM, but the *en face* OCT angiographic information is also available from the same scan to detect occlusive vascular events.

Previously, it was thought that arteriolar and globular subtypes of PAMM resulted from pre-capillary arterial ischemia with the arterial occlusions then leading to downstream infarction of the intermediate and deep capillary plexuses, resulting in more wide-spread patterns of ischemia.^4^^,^^7^ In contrast, it was thought that the peri-venular fern-like PAMM resulted from sluggish capillary flow and ischemia that affected the venous side of the capillary system.^1^^,^^7^^,^^8^ It was believed that in events involving venous insult, ischemia could occur more easily in the DCP because this region is a watershed-like region, leading to the fern-like distribution of PAMM seen in retinal vein occlusions.^1^^,^^2^^,^^9^^,^^10^ In the mildest forms, it is thought that ischemia occurs at the venous end of the DCP leading to perivenular PAMM that may evolve laterally to become more diffuse^11^ When severe progression occurs such as in globular PAMM, ischemia follows a vertical ascent to involve the inner retina.^11^

In addition to our report, Bakhoum et al. also reported on patients with retinal artery occlusions showing a peri-venular fern-like PAMM pattern on en face structural OCT imaging.^12^ All these cases demonstrate that peri-venular PAMM can occur in the setting of any type of ischemic event. Patients with this peri-venular fern-like pattern of PAMM exhibit better final visual acuity compared with those with globular pattern.^12^ All three of our patients had impressive recoveries in visual acuity with resolution of most of the PAMM-like changes on *en face* structural OCT images despite having fundus findings consistent with retinal arterial occlusions. Thus, it is not the condition that defines the pattern of PAMM, but more likely, the severity of the ischemic event. Perivenular PAMM may be an important biomarker of retinal arterial and venular occlusions with milder severity that may or may not progress over time.

## 4 Conclusions

The peri-venular fern-like pattern of PAMM can be detected on *en face* structural OCT images when FA fails to detect underlying vascular abnormalities. The *en face* images of the middle retina from the 12 × 12 mm scans are a useful tool for detecting a wider area of involvement for this peri-venular ischemia. The fact that the peri-venular fern-like patterns of PAMM can be seen in conditions such as arterial occlusions suggests that this pattern is a common ischemic pathway for all vascular ischemic conditions, with the severity of the vascular event predicting the pattern of PAMM.

## Patient consent

Consent to publish this case report has been obtained from the patient(s) in writing.

## Funding/support

Research supported by grants from Carl Zeiss Meditec, Inc. (Dublin, CA), the Salah Foundation, an unrestricted grant from the Research to Prevent Blindness, Inc., New York, NY, and the National Eye Institute Center Core Grant (P30EY014801) to the Department of Ophthalmology, University of Miami Miller School of Medicine. The funding organizations had no role in the design or conduct of this research.

## Financial disclosures

Dr. Gregori and Dr. Rosenfeld received research support from 10.13039/501100002806Carl Zeiss Meditec, Inc. Dr. Gregori and the University of Miami co-own a patent that is licensed to Carl Zeiss Meditec, Inc.

Dr. Rosenfeld also receives additional research funding from Stealth BioTherapeutics. He is also a consultant for Apellis, Biogen, Boehringer-Ingelheim, Carl Zeiss Meditec, Chengdu Kanghong Biotech, EyePoint, Ocunexus Therapeutics, Ocudyne, and Unity Biotechnology. He also has equity interest in Apellis, Valitor, Verana Health, and Ocudyne.

Dr. Swaminathan is a consultant for Sight Sciences.

The following authors have no financial disclosures: PGI, OT, YS, MS, MK.

## Authors

All authors attest that they meet the current ICMJE criteria for Authorship.

## References

[1] Rahimy E., Sarraf D., Dollin M.L., Pitcher J.D., Ho A.C. (2014). Paracentral acute middle maculopathy in nonischemic central retinal vein occlusion. Am J Ophthalmol.

[2] Chen X., Rahimy E., Sergott R.C. (2015). Spectrum of retinal vascular diseases associated with paracentral acute middle maculopathy. Am J Ophthalmol.

[3] Sridhar J., Shahlaee A., Rahimy E. (2015). Optical coherence tomography angiography and en face optical coherence tomography features of paracentral acute middle maculopathy. Am J Ophthalmol.

[4] Sarraf D., Rahimy E., Fawzi A.A. (2013). Paracentral acute middle maculopathy: a new variant of acute macular neuroretinopathy associated with retinal capillary ischemia. JAMA Ophthalmol.

[5] Ghasemi Falavarjani K., Phasukkijwatana N., Freund K.B. (2017). En face optical coherence tomography analysis to assess the spectrum of perivenular ischemia and paracentral acute middle maculopathy in retinal vein occlusion. Am J Ophthalmol.

[6] Garrity S.T., Tseng V.L., Sarraf D. (2018). Paracentral acute middle maculopathy in a perivenular fern-like distribution with en face optical coherence tomography. Retin Cases Brief Rep.

[7] McLeod D., Beatty S. (2015). Evidence for an enduring ischaemic penumbra following central retinal artery occlusion, with implications for fibrinolytic therapy. Prog Retin Eye Res.

[8] Iafe N.A., Onclinx T., Tsui I., Sarraf D. (2017). Paracentral acute middle maculopathy and deep retinal capillary plexus infarction secondary to reperfused central retinal artery occlusion. Retin Cases Brief Rep.

[9] Yu S., Wang F., Pang C.E., Yannuzzi L.A., Freund K.B. (2014). Multimodal imaging findings in retinal deep capillary ischemia. Retina.

[10] Philippakis E., Dupas B., Bonnin P., Hage R., Gaudric A., Tadayoni R. (2015). Optical coherence tomography angiography shows deep capillary plexus hypoperfusion in incomplete central retinal artery occlusion. Retin Cases Brief Rep.

[11] Scharf J., Freund K.B., Sadda S., Sarraf D. (2020). Paracentral acute middle maculopathy and the organization of the retinal capillary plexuses. Prog Retin Eye Res.

[12] Bakhoum M.F., Freund K.B., Dolz-Marco R. (2018). Paracentral acute middle maculopathy and the ischemic cascade associated with retinal vascular occlusion. Am J Ophthalmol.

